# Sustainability and food security after COVID-19: relocalizing food systems?

**DOI:** 10.1186/s40100-020-00167-z

**Published:** 2020-09-17

**Authors:** Walter Belik

**Affiliations:** Institute of Economics, University of Campinas (Unicamp), Rua Pitágoras, 353 CEP 13083-857 Campinas, São Paulo, Brazil

Considering the current pandemic, in which changes in the food system and the relationship between food and society are under discussion, I take the opportunity with this editorial to offer some points for debate.

Undoubtedly, the most striking feature of this pandemic regarding food-related issues is the growing prevalence of malnutrition across the world. It is well known that countries have not been able to achieve the goals set by the 1996 World Food Summit (halving the number of malnourished people by 2015 - based on the year 1992), nor the goals of the 2000 Millennium Summit (halving the percentage of malnourished people). It is also clear by now that countries will not be able to comply with the Sustainable Development Goals signed in 2015, such as ending hunger and all forms of malnutrition by 2030, among other commitments. Since the financial crisis that lasted until 2010, the number of malnourished people is no longer decreasing, ending a trend that had been going on since the end of the 1990s. Looking at the last decade, worldwide results are disappointing and there are indications that the situation will become even worse.

However, what the course of the pandemic is showing so far is that the increase in malnutrition is not occurring because of a lack of food. On the contrary, production remains high, but there is a reduction in the demand for food caused by the interruption of work and entertainment activities such as hotels, restaurants, schools, and industrial facilities. International food trade also suffered due to logistic difficulties and higher sanitary barriers. All of this has been causing an oversupply of food, leading to greater food losses and waste.

The COVID-19 crisis is expected to throw millions of workers into unemployment. ILO estimates a loss of 300 million full-time jobs in terms of hours worked (ILO Monitor, 2020). The same organization also predicts that the 2 billion informal workers across the globe will be the ones most affected by the crisis. The loss of jobs and income will be directly reflected in poverty indicators. According to the World Bank, the share of the global population in extreme poverty (living on a per capita monthly income less than US $ 1.90 PPP a day) that had fallen below 10% is now expected to rise again, and a contingent between 71 and 100 million people will return to the extreme poverty condition (Mahler et al. 2020). Globally, the IMF forecasts a 4.9% decline in the GDP for the year 2020 (World Economic Outlook (WEO) 2020). This decline will be felt most intensely in emerging exporting countries. In Latin America, for example, ECLAC (United Nations Economic Commission for Latin America) predicts that the region should return to the economic situation of 10 years ago (CEPAL 2020).

As mentioned, these pessimistic figures will have a direct impact on the food security and nutrition indicators, moving us further away from the Sustainable Development Goals. FAO (UN Food and Agriculture Organization) releases its malnutrition data in 3-year averages and does not yet make predictions for the present year. However, the WFP (World Food Program), a UN agency directly involved in combating hunger, estimates that 265 million individuals will be in acute food insecurity compared to the 135 million before the crisis, and hence, 820 million individuals worldwide will suffer from hunger by the end of 2020 (Blog WFP 2020).

This context of crisis may offer an opportunity to introduce new questions on the habits and patterns in the Global Food System. It may be expected that the pandemic has weakened the Global Food System and showed its vulnerability and dependence in relation to activities such as long-distance transport, processing of raw materials and packaging. In this sense, new practices and the possibility of re-connecting the countryside, where food production occurs, with the consumption side is a real one, and can become concrete. Moreover, in the face of the pandemic and considering the changes resulting from new international economic frameworks, is there room for the growth of food sovereignty movements?

Evidence suggests that the international trade will shrink this year and in the years to come. Information from the WTO shows that, since the start of the pandemic in the first months of 2020, most of maritime and air transport was interrupted, and there is no prospect of normalization of these activities in the short term (World Trade Organization information note 12 August 2020, 2020). In the case of food distribution, this disruption in global supply chains has two immediate negative effects: it destroyed jobs in producing and exporting countries and caused shortages in certain consumer markets.

In general, due to the neoliberal wave, countries have already been reducing the volume of strategic, regulatory food reserves while specializing their production in search of comparative advantages. Thus, with the interruption of trade flows, countries have been forced to return to local producers and accumulate stocks of the products that are most prevalent in the diet of their populations.

Nevertheless, local production does not translate directly or automatically in a local purchase. The re-connection of field-to-fork also depends on the functioning of local distribution and marketing structures. In the case of wholesale food structures, surveys conducted in Latin America and the Caribbean show that these markets witnessed only a slight reduction in supply, which was attributed to seasonality more than to product availability (FAO-FLAMA Newsletters 2020). On the other hand, regarding the case of direct distribution, local fairs were closed down and regional markets reduced their activities due to health problems. Sales of organic products, which had been growing at high rates before the crisis, have been reduced to a minimum due to difficulties in marketing. Direct online sales connecting consumers and producers have been spreading quickly, but the effects are still insipient. Online transactions by applications have not gone beyond the common practices of relationships between producers and consumers, as was already being practiced in face-to-face terms by CSAs (Community Supported Agriculture), for example.

Although the habit of cooking at home has expanded, it is not clear whether quarantine and social isolation have improved the population’s standards of nutrition. On the contrary, at least part of society has fallen into poverty and consequently reduced consumption, while the other part may have switched to convenience foods either when buying or preparing their meals.

Both the dominance of the food industry and large supermarket chains and the reduction in the activities of the wholesale centers for fresh products, local markets and open markets, have put the local food producer at a disadvantage towards the supply of processed and ultra-processed foods. Under the conditions of this new food environment created by the pandemic, the ways of commercialization have changed and the uncertainty regarding prices and sanitary conditions in transport has increased. All this has had a devastating impact on family farming, specialty producers, artisanal fishermen and seeds, and fruit and plant collectors’ activities.

Measures to counter the pandemic, such as social distancing, are expected to last in the medium term and have already led to changes in purchasing habits. The main impact of these new conditions is likely to resonate in small- and medium-sized food retail businesses and open markets that are unable to adapt to the new reality of contact-free and online purchases. In China, where this system is spreading rapidly due to the official policy of extinguishing street commerce (Nong Gai Chao), supermarkets are rapidly gaining ground and taking over the coordination of value chains.

In contrast, even among the low-income population, other tools for direct purchases of organic, certified, or local foods are shyly emerging. The use of online apps is still growing, but also solidarity purchases and other creative forms of exchange are expanding, helping to link producers to consumer groups. The use of these new arrangements requires some type of external organization or coordination, which could be supported by an NGO or cooperative, to reinforce the most complicated link in the supply chain, namely door-to-door logistics and distribution.

From the government side, new tasks are proposed for public policy in the post-COVID period. The pandemic demonstrated how fragile global supply chains are. Local administrations have several challenges to address. In addition to the obligation to combat malnutrition and the increase in obesity, there is a tendency for new food deserts to be formed, which will require greater attention in urban regulatory elements, for example. Food aid programs engaging local producers and government purchases are also expected to be strengthened. In this context, the challenge of public policies involves a greater effort in planning the development of logistics platforms and distribution centers based on international health standards, but that can still preserve the uniqueness of local culture, knowledge, and traditions of food production and consumption.
